# A neuro-symbolic approach to translate English to logic and ontology

**DOI:** 10.3389/frai.2026.1820380

**Published:** 2026-08-07

**Authors:** Adam Pease, Richard Thompson

**Affiliations:** Computer Science Department, Naval Postgraduate School, Monterey, CA, United States

**Keywords:** large language models, logic, machine learning, ontology, theorem proving

## Abstract

Human language is often vague and ambiguous. There have been many efforts to create formal languages and many attempts to translate human language into formal languages. Logic has a great deal of flexibility, not least in how the symbols used are defined. We anchor lexical elements in a formal ontology, which helps ensure that the meaning of symbols is stable across discourse. We generate a large corpus of language and logic pairs that are used to train a machine learning system. The training data is generated algorithmically from our ontology. Since even a very large training corpus cannot capture all the possible sentences in human language, we employ large language models to simplify language and make it more likely that input sentences will be similar to forms that are in the training data.

## 1 Introduction

Human language is often vague and ambiguous. There have been many efforts to create formal languages and many attempts to translate human language into them. Logic has a great deal of flexibility. If a common and formally defined set of constant terms is not used, the same thought can be expressed in many different ways. We anchored lexical elements in a formal ontology, which helps ensure that the meaning of symbols remains stable across discourse. We generated a large corpus of language and logic pairs that are used to train a machine learning system (Pease et al., 2023; Thompson et al., 2025a). The training data is generated algorithmically from our ontology. Since even a very large training corpus cannot capture all the possible sentences in human language, we used a large language model to simplify language and make it more likely that input sentences will be similar to forms that are in the training data (Milanese et al., 2025; Singley et al., 2025).

## 2 Background: ontology

Human language is very flexible. Different languages can have symbols “table,” “mesa” or 桌子 and mean the same thing. Speakers of the same language can use “spanner” and “wrench” and mean the same thing. In propositional logic, the letter “a” could just as easily stand for “It is raining.” as “John likes Sue.” In first order logic, one could use a predicate “has” to mean variously “possesses,” “owns” or that something exhibits some attribute or quality.

A controlled vocabulary can be used to constrain some of the flexibility of natural language. But that still requires some tacit agreement and shared background among users of that language to ensure that there is mutual understanding of the symbols.

An ontology (Pease, 2026) aims to capture the meaning of symbols in an objective and computable way. The extent to which it does so depends on the expressive power of the formal language used (Pease, 2021). An ontology that captures only terms and relationships in a graph captures some of the ways we describe the world, but there are many things we say are more complex than a simple binary relationship.

Products labeled as ontologies vary widely in complexity, but most are not written in expressive logics. Some upper ontologies are quite small, with a few dozen to a hundred concepts. These include BFO (Otte et al., 2022) and DOLCE (Gangemi et al., 2002). These have been extended by large terminological hierarchies that use less expressive logics such as Description Logic (Baader et al., 2007).

We used the Suggested Upper Merged Ontology (SUMO)^1^ in this study. SUMO is a large-scale formal ontology with approximately 20,000 concepts and 100,000 human-authored logic statements (Niles and Pease, 2001; Pease, 2011), encompassing numerous specialized domains (Pease and Benzmüller, 2010). It includes over 15,000 types of physical objects, 1,425 process types, hundreds of social roles, and over a thousand relationships, each axiomatically defined in first- and higher-order logic. The Sigma Knowledge Engineering Environment (Pease and Schulz, 2014) enables translation into TPTP-family languages (Sutcliffe, 2010): First Order Form (FOF) (Trac et al., 2008), Typed First-order form with Arithmetic (TFA) (Pease, 2023), and Typed Higher-order form (THF) (Benzmüller and Pease, 2010, 2012), for automated reasoning with provers such as Vampire (Kovács and Voronkov, 2013) and Eprover (Schulz, 2002).

SUMO is stated in the SUO-KIF language^2^ which has a simple syntax based on Lisp S-expressions. It uses prefix notation in which the six standard logical operators, plus equality, are the only primitives: and, or, not, exists, forall, =>, < =>, =, and they have the usual first-order semantics. Variables are indicated with a leading “?” character. Quantifiers are followed by a list of quantified variables and then the formula that is within their scope. Unquantified variables are implicitly universally quantified. Parentheses are used for grouping and precedence and whitespace as delimiters.

The translators from SUMO to TPTP languages implement a type system on top of untyped logics, using the relation type restrictions specified in SUMO to create type guards on formulas (Pease and Schulz, 2014). The use and translation to first-order logic of predicate variables (variables in the predicate position) and row variables (variables that stand for one or more arguments to a relation) allow ontologies to specify general formulas that would otherwise be unsupported by the syntax of first-order logic. This allows, for example, stating the axiom of transitivity once and having it apply to hundreds of transitive relations. In the translation of SUMO to TFA, numbers and arithmetic operations are supported, which is rare in purely declarative knowledge representation languages, yet such a facility is essential for representing a large portion of common linguistic utterances. Statements involving numbers and arithmetic are absent in most ontologies and knowledge representation efforts. People commonly talk about times and dates such as “...a week from now...,” “...after January 7th...,” “...by 4 pm...” or counts and measures “...5 chairs...,” “...3 gallons...” so this must be part of any attempt to create a comprehensive representation of language in a logic. For example, the incubation time of tetanus is 3–21 days (Listing 1), and the height of water on any given day at high tide is greater than that at low tide (Listing 1). For comparison, Listing 2 expressed in traditional logic notation and abbreviating the predicate and function names would be


$$\begin{array}{l} \forall P,T1,T2,A1,A2,U,D:\\ \;(lt(P,T1,m(A1,U))\;\wedge \\ \;ht(P,T2,m(A2,U))\;\wedge \\ \;i(U,unit\;Of\;Length)\;\wedge \\ \;i(D,day)\;\wedge \\ \;ot(T1,D)\;\wedge \\ \;ot(T2,D))\to \\ \;(A2>A1)) \end{array}$$

Listing 1 Tetanus Incubation


(diseaseIncubation Tetanus 
   (MeasureFn 3 DayDuration)
   (MeasureFn 3 WeekDuration))

Listing 2 High Tide


(=>
   (and
      (lowTide ?PLACE ?TIME1
          (MeasureFn ?AMOUNT1 ?U))
      (highTide ?PLACE ?TIME2
          (MeasureFn ?AMOUNT2 ?U))
      (instance ?U UnitOfLength)
      (instance ?DAY Day)
      (overlapsTemporally ?TIME1 ?DAY)
      (overlapsTemporally ?TIME2 ?DAY))
   (greaterThan ?AMOUNT2 ?AMOUNT1))

Early in the SUMO project (Niles and Pease, 2003), we manually linked each of the 117,000 word senses in the WordNet lexico-semantic database (Fellbaum, 1998) to the concepts in SUMO. Each word sense consists of a numerical identifier (or synset id) and a short definition or “gloss.” Word senses were given an equivalent link to a SUMO term if that term was roughly equivalent to the WordNet word sense. If there was no equivalent term, then a subsuming mapping to the SUMO term was made. In subsequent years, as SUMO has grown, approximately 40,000 senses were remapped to newer, more specific SUMO terms. For example, WordNet contains five senses for the word “rail.”

- 104046810 short for railway; “he traveled by rail;” “he was concerned with rail safety.” SUMO Mappings: Railway (equivalent mapping).
- 104463679 a bar or pair of parallel bars of rolled steel making the railway along which railroad cars or other vehicles can roll. SUMO Mappings: RailroadTrack (equivalent mapping).
- 104046590 a horizontal bar (usually of wood or metal). SUMO Mappings: Artifact (subsuming mapping).
- 102014941 any of numerous widely distributed small wading birds of the family Rallidae having short wings and very long toes for running on soft mud. SUMO Mappings: Bird (subsuming mapping).
- 104047401 a barrier consisting of a horizontal bar and supports. SUMO Mappings: Handle (subsuming mapping).

WordNet also contains links among its word senses, including synonym links. For example, the word sense of ‘rail' equivalent to SUMO's RailroadTrack has the synonyms in WordNet: “rail,” “rails,” “runway,” “track.” To each of these, we added a link to the most closely related SUMO concept. In some cases, concepts are equivalent or nearly so to the English word sense, as with Railway and RailroadTrack. In other cases, such as for the small bird called a “rail,” we only have a more general term for Bird in SUMO. In addition to “equivalent mapping” and “subsuming mapping,” there is a third mapping type, “instance mapping,” in which a general SUMO term maps to a proper noun in WordNet, as in the “Battle of Gettysburg.” Notably, these relations are necessarily approximate and informal, because they involve a relationship to informal natural language, in contrast with the mathematically-defined symbols in SUMO.

- 101279615 a battle of the American Civil War (1863); the defeat of Robert E. Lee's invading Confederate Army was a major victory for the Union. SUMO Mappings: Battle (instance mapping).

Note that SUMO has been built entirely independently of WordNet, and the mapping has been done after SUMO concepts were created.

In this study, we adopt a typographical convention that SUMO terms will be in typewriter font. These identifiers can be entered into the “KB term” field in the online SUMO browser^3^ to view their complete definition.

## 3 Related work: language to logic translation

There is a significant body of study on natural language-to-logic translation. Foundational studies began with controlled English and narrow domains. These early studies include ACE (Fuchs and Schwitter, 1996) and CELT (Pease and Murray, 2003; Pease and Li, 2010). TRAINS was an effort to create a “planning assistant” that would attempt to understand human speech and parse out meaning (Allen et al., 1995). These early projects did not leverage Large Language Models, and only CELT employed a large general ontology.

LogAnswer was an early attempt at a question-and-answer system (Furbach et al., 2008) with the help of a large semantic network. It used substantial portions of the MultiNet semantic network (Helbig, 2005) and could answer basic queries. Other more recent efforts have used LLMs to turn natural language into formal logic (Pan et al., 2023; Zheng et al., 2025; Noble et al., 2025; Sutcliffe, Personal communication^4^). Each of these studies takes natural language and prompts an LLM for a formal logic representation, which is then fed into an automated proving system. While important, these studies perform translation in the absence of an ontology, and their terms are not grounded in concepts outside the prompt contexts. This means that semantically equivalent utterances can wind up with syntactically different and unrelated formalizations. Absent the background knowledge from a large ontology, symbols have meaning only through their use in those utterances, whereas a critical portion of human understanding is based on a shared background of pragmatic information about the symbols exchanged.

Frame structures have long been used to generate English/Logic pairs from ontologies (Karp, Peter D, 1992), but not on the scale of our research (millions of sentences) in an expressive logic. Recently, researchers have developed a system to translate natural language into first-order logic using an LLM (Tammet et al., 2024), including deploying LLMs to generate a synthetic training dataset of 34,000 sentence/logic pairs (Yang et al., 2023), with limited linguistic and logical complexity.

Among the more relevant studies attempting to add formalism to AI-generated content was one conducted by Facebook AI researchers with a chatbot called bAbI (Weston et al., 2016). Like many statistical AI systems, this project provides natural-sounding responses but also uses a limited ontology of a few dozen labels to add some meaning and reasoning to certain responses.

One characteristic of these prior studies, other than CELT, which was implemented with SUMO, is the absence of a library of formalized concepts. Without definitions to anchor the meaning of terms used in logic expressions, symbols have meaning only to the extent that they exist in the current dialogue. Without additional training, LLMs can now generate logical forms from English, but they do not capture the semantics of labels because the formalizations do not align with the semantics people would assign to those labels. For example, “The party is on Thursday.” and “The book is on the table.” both feature the preposition “on” but with a completely different meaning. Without an ontology to anchor the meaning of the logical symbols, contradictions can easily be generated.

Abstract Meaning Representation (AMR) is a natural language representation framework using directed graphs (Banarescu et al., 2013). Numerous successful applications and use cases exist for AMR (Tohidi and Dadkhah, 2022); however, it is not designed to support formal logical inference, and thus is limited by a lack of a formally defined typing system, fully explicit quantification, concept inheritance, or a proof theory.

Logical Inference via Neurosymbolic Computation (LINC) (Olausson et al., 2023) is another language-to-logic system that uses large language models to transform English into first-order logic. It does not address how to connect the formalizations to an existing large symbol set, or how to ensure that sentences with the same semantics but different phrasing or word choice generate the same logical formulas.

## 4 Materials and methods

**Figure 1:**
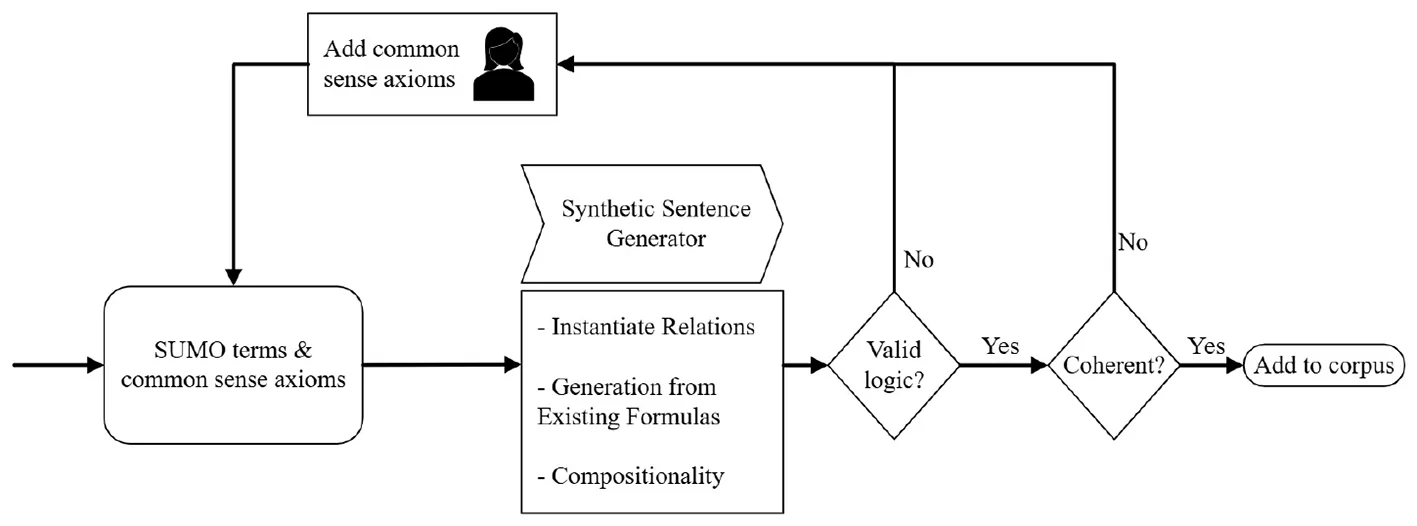
Synthetic sentence generation flowchart.

Our system generates language-logic pairs designed for training a machine learning system. The general generation method is shown in Figure 1. In each approach, English words, formulas, and logic terms are taken from the SUMO ontology. Several approaches were used:

1. *Instantiate Relations*- instantiate relations with arguments of appropriate types and then generate natural language (NL) paraphrases from them (Section 4.1).
2. *Generate from Existing Formulas*- iterate through all formulas in SUMO and generate NL paraphrases (Section 4.2).
3. *Compositionality*- build up sentences and logic expressions compositionally (Section 4.3).

After generation, the logic formula is checked for syntactic and semantic validity. If valid, the English sentence is checked for coherence as described in Section 4.7. Accepted English-logic pairs are then added to our corpus. If the logic expression is invalid or the English sentence is incoherent, then the English-logic pair is stored for later analysis by a trained ontologist, who determines if a SUMO type restriction or axiom can be added to the ontology to prevent future errors in generation. This will be discussed in greater detail in Section 4.6.

### 4.1 Instantiate relations

One of the simplest procedures to generate language logic pairs is to use the languages templates that have been created for SUMO's relations. There are approximately 1,800 different relations in SUMO, the majority are binary, but many take three or more arguments. Each has a language paraphrase template that uses a simple format similar to C-language printf statements. This process is visible in the paraphrases that are generated in the right-hand column when browsing any SUMO term in the online Sigma browser, for example, Object^5^. For example, the geographicSubregion relation relates two instances of GeographicArea to say that the first argument is part of the second. We have that Kenya is part of EasternAfrica (as well as facts that Nations are LandAreas which in turn are GeographicAreas (Listing 3).

Listing 3 Geographic Areas


(instance Kenya Nation)
(termFormat EnglishLanguage Kenya ~Kenya~)
(instance EasternAfrica GeographicArea)
(termFormat EnglishLanguage EasternAfrica ~Eastern␣Africa~)
(geographicSubregion Kenya EasternAfrica)

We have the following linguistic data that has been created by hand when the SUMO relation was specified (Listing 4). The Sigma system replaces “%1” with “Kenya” and “%2” with “Eastern Africa” to generate “Kenya is a geographic subregion of Eastern Africa.” The characters “%n” would be replaced by “not” if the formula were negated.

Listing 4 Geographic Subregion


(format EnglishLanguage geographicSubregion 
~%1␣is␣%n␣a␣geographic␣subregion␣of␣%2~)

The relation paraphrase templates must be manually created because the appropriate phrasing and even the order of arguments may differ in a colloquial reading of different relations. For example, the relation of victim has the template shown in (Listing 4) where the arguments have the reverse order from our first example (Listing 6), in which the positive and negative phrasings require additional detail.

Listing 5 Simple Formatting Statement


(format EnglishLanguage victim ~%2␣is␣the␣victim␣in␣%1~)

Listing 6 A “format” expression


(format EnglishLanguage externalDebtInPeriod ~%1␣%p{is} 
␣␣%n{isn't}␣external␣debt␣in␣period␣%2␣for␣%3~)

All relations have a defined type signature. For example, (Listing 7), which states that the relation part requires two instances of the type Object as arguments, and that geographicSubregion requires instances of GeographicArea. Knowing those types provides some information on what entities might be used to generate new sentences. There are 15,000 subclasses of Object in SUMO. 11,000 of those are under Artifact which is “An Object that is the product of a Making.” or more formally, “If there is an artifact, then there exists a process of making that resulted in that artifact.” Each of these identifiers -Making, result—have their own logical definition (Listing 8).

Listing 7 Type Restrictions


(domain part 1 Object) 
(domain part 2 Object)
(domain geographicSubregion 1 GeographicArea)
(domain geographicSubregion 2 GeographicArea)

Listing 8 An Axiom on Artifact


(=>
     (instance ?ARTIFACT Artifact)
     (exists (?MAKING)
        (and
              (instance ?MAKING Making)
              (result ?MAKING ?ARTIFACT))))

Were we to randomly pick just the first two artifacts shown^6^ (Listing 9) and use the part relation, we could generate (part APiston AnEngineCylinder) and the sentence “A piston is a part of an engine cylinder.” A difficulty in generating coherent sentences occurs when selecting terms. If selecting terms randomly, we could have easily picked a different two subclasses of Artifact, such as EngineCylinder and Chain, resulting in the sentence “An engine cylinder is part of a chain.” It is hard to imagine an engine cylinder being (literally) part of a chain. The generated English-logic pair would be syntactically correct while semantically nonsense. Type restrictions and other axioms can be added to SUMO to ensure that each logic expression is correct and the English is more coherent. In the latest run of the system before this study, out of a set of around 850,000 generated English-logic pairs, 2.2% contained logic errors. These errors are removed from the database and continuously analyzed to improve future sentence generation.

Listing 9 Artifacts


(subclass Piston Artifact) 
(subclass EngineCylinder Artifact)

Although type restrictions are part of the solution, they are not enough. We will return to this issue later in the chapter.

### 4.2 Generation from existing formulas

The second approach to generating sentences is relatively straightforward, given the logic-to-language generation code already in place. We run through every logical statement in SUMO and generate a sentence for each. For example, the simple logic statement in (Listing 10) has the auto-generated English equivalent “A bear is a subclass of carnivore.” Because those statements have been created by humans and further verified against each other with automated theorem proving, we can have confidence that they are not nonsense. With 100,000 formulas in SUMO, that is already a substantial training set. However, there is a different problem that this does not form a *balanced corpus* (Chen et al., 1996). It is heavily biased toward simple factual statements and rules that define concepts. Much of human writing and dialogue is about narratives of events, and that is conspicuously missing from the set of existing SUMO formulas.

Listing 10 A subclass Statement


(subclass Bear Carnivore)

### 4.3 Compositionality

To create novel language-logic pairs not directly derived from a complete logical statement, we attempt to build up sentences from smaller components and combine them. The process of building sentences and their logical equivalents begins with random selection from SUMO's approximately 1,500 Process types.

English is a Subject-Verb-Object language. We assume that basic structure for sentences, but also have the possibility to perturb it. If the Process chosen is an IntentionalProcess then the subject of the sentence must be a common name of a Human, an AutonomousAgent (such as a Nation or an Organization) a SocialRole (such as Employee or Plumber), or a pronoun such as “You” or “Who.”

WordNet has a set of *verb frames*^7^ that constrain the subject and object of verbs. Since we have mappings from all SUMO terms from WordNet, once we have a Process, we can get the WordNet identifier and find the corresponding verb frame. For example,

*Verb Synset*: 202230772*SUMO Mappings*: Giving (subsuming mapping)*Words*: give, hand, pass, pass_on, reach, turn_over*Gloss*: place into the hands or custody of; “hand me the spoon, please;” “Turn the files over to me, please;” “He turned over the prisoner to his lawyers.”

This verb has the following sequence of codes in the WordNet verb inventory 09 00 + 14 00 + 15 00 signifying that verb frames 9, 14, and 15 each apply to “00,” which means all the words in the set of synonyms, so “give, hand, pass...” etc. Looking at frame 15 we have

15 Somebody —-s something to somebody

This constrains the sentence generation to make the subject and indirect objects humans. However, this is not sufficient to eliminate nonsense sentences.

On top of this basic syntactic structure are layered many other common linguistic constructs, including:

- Indirect objects.
- Tenses for the verbs.
- Modals.
- Counts and measures of things.
- Social and job roles, as well as the names of people.
- Negations, questions.

#### 4.3.1 Indirect objects

SUMO utilizes Davidsonian semantics (Davidson, 1967) for action sentences, in which verbs are treated as instances of Process types and the entities that participate in those processes are related via CaseRoles. For example, “Doug massages the plumber.” uses the SUMO Process type of Massaging and the CaseRoles of agent and patient (Listing 11).

Listing 11 A Correct if Curious Sentence


(exists (?S ?V ?O)
  (and
    (instance ?S Human)
    (names ~Doug~ ?S)
    (instance ?V Massaging)
    (instance ?O Human)
    (attribute ?O Plumber)
    (agent ?V ?S)
    (patient ?V ?O)))

Indirect objects are more challenging since they depend upon both the verb and the types of the subject and object. WordNet's sentence frames provide a few different restrictions as follows prepositions appear: “to,” “on,” “from,” “with,” “of,” “that,” “into,” and “whether.” These correspond to the case roles, which is mentioned in Table 1.

**Table 1:** Indirect object prepositions and their SUMO case roles.

Preposition	SUMO case role
“to”	destination
“from”	origin
“with”	instrument
“of”	patient
“on”	destination

However, there are 68 case roles in SUMO^8^ and we will make much more complete use of them in a future version of this study. For example, “John argues for his client.” could use the attorney case role if the dialog context identifies “John” as an attorney (Listing 12).

Listing 12 Arguing


(exists (?J ?A ?C) 
  (and 
    (instance ?J Human)
    (instance ?A Arguing) 
    (instance ?C Human) 
    (names ~John~ ?J) 
    (attorney ?A ?J) 
    (benefits ?A ?C)))

In a related project, we are working on spatial representations of visual knowledge. This entails building sentences that make use of SUMO's PositionalAttributes (such as Above, East, Inside etc.) and SpatialRelations, (such as traverses, connects, partiallyFills etc).

#### 4.3.2 Tenses

In our generated L2L training set, we used the past, present, and future tenses, as well as progressives, for each. These are all expressed formally with respect to a deictic “Now” (Stojnić and Altshuler, 2021). SUMO captures the functional relationship between processes and the time at which they occur with the function WhenFn. For example, “John walked.” has the corresponding formula shown in (Listing 13).

Listing 13 Walking


(exists (?J ?W) 
  (and 
    (instance ?J Human) 
    (names ~John~ ?J) 
    (instance ?W Walking) 
    (earlier 
  (WhenFn ?W) 
  Now)))

SUMO has the thirteen Allen relations between temporal intervals (Allen, 1983), as well as terms to express metric times and dates, holidays and anniversaries. “John walked at 2:43 pm on March 13, 2006” is formalized as (Listing 14).

Listing 14 Walking at a Time and Date

 
(exists (?J ?W) 
  (and 
    (instance ?J Human) 
    (names ~John~ ?J) 
    (instance ?W Walking) 
    (during 
      (WhenFn ?W) 
      (MinuteFn 43 
        (HourFn 14 
          (DayFn 13 
              (MonthFn March 
                (YearFn 2006))))))))

Notably, clarity and space reasons in the remaining examples in this paper, we will remove the tense expressions.

#### 4.3.3 Modals

SUMO supports quantified multi-modal logic with Kripke semantics (Pease, 2025) including automated translation to the TPTP TFF and THF languages for reasoning in Vampire. We have the epistemic and epistemic-like relations of knows, believes, says, and desires. We used the standard alethic modalities of Necessity and Possibility. We have the deontic modalities of Obligation, Permission, and Prohibition. While considerable further research remains to be done to achieve consistent reasoning in a quantified multi-modal logic, it is essential to note that these are not merely labels but are axiomatized using Kripke's accessibility relations (Ballarin, 2023) and different modal systems appropriate for each modality. We are using S4 or S5 for epistemic operators, KD45 for doxastic logic and system D for deontics. “John knows that Mary walks to the store.” is expressed logically as (Listing 15)

Listing 15 Epistemic Expression


(exists (?J) 
  (and 
    (instance ?J Human)
    (names ~John~ ?J)
    (knows ?J
      (exists (?M ?W ?S)
        (and
          (instance ?M Human)
          (names ~Mary~ ?M)
          (instance ?W Walking) 
          (instance ?S RetailStore) 
          (agent ?W ?M) 
          (destination ?W ?S))))))

#### 4.3.4 Counts and measures of things

SUMO includes 2,000 types of CorpuscularObjects and over 900 substances, corresponding to the Aristotelian object-substance distinction (Robinson and Weir, 2024). Objects can have counts, and both objects and substances can have associated measures. We ensured that phrases such as “5 gallons of water” or “three chairs” are supported. For example, “19.11 liters of alcoholic beverage will be fermenting.” (Listing 16)

Listing 16 A Measure


(exists (?A ?F) 
  (and 
    (instance ?F Fermenting) 
    (instance ?A AlcoholicBeverage) 
    (patient ?F ?A) 
    (measure ?A 
      (MeasureFn 19.11 Liter))))

SUMO has over 300 units of measure encompassing the System International units, Imperial (English) units, and many others.

### 4.4 Social and job roles as well as names of people

There are 340 social roles in SUMO, such as Athlete, Manager, and Mayor. In SUMO, classes are *rigid* and therefore do not change over the life of an object (LaPorte, 2022). Social roles are attributes or qualities that may change—an individual might be a professor during the week but be a plumber on the weekend when a faucet breaks. However, if that individual ceases to be a human, then he or she no longer exists. For example, “The tourist interviewed Marilyn.” is formalized as someone has the attribute of being a tourist (Listing 17).

Listing 17 Social Role


(exists (?T ?I ?M)
  (and 
    (attribute ?T Tourist) 
    (instance ?I Interviewing) 
    (instance ?M Human) 
    (names ~Marilyn~ ?M) 
    (agent ?I ?T) 
    (patient ?I ?M)))

#### 4.4.1 Negations and questions

Sentences can be negated at several points and in several different ways. A very minor change to the sentence can result in a very different formalization. “John didn't walk to the store.” means there is a particular store that John didn't walk to (Listing 18) “John didn't walk to a store.” means there is a particular walking done by John, where a store was not the destination (Listing 19).

Listing 18 Negation 1


(exists (?J ?S)
  (and
    (instance ?J Human) 
    (names ~John~ ?J) 
    (instance ?S RetailStore)
    (not (exists (?W) 
      (and 
        (instance ?W Walking)
        (agent ?W ?J) 
        (destination ?W ?S))))))

Listing 19 Negation 2


(exists (?J ?W) 
  (and 
    (instance ?J Human) 
    (names ~John~ ?J) 
    (agent ?W ?J) 
    (instance ?W Walking) 
    (not 
      (exists (?S) 
        (and 
          (instance ?S RetailStore) 
          (destination ?W ?S))))))

Negations can be placed before modals, as in “John doesn't know that Mary walked to the store,” as well as within their scope “John knows that Mary didn't walk to the store.”

We can generate questions (including question words such as “who”) such as “Who went to the store?” and “Did John go to the store?” and combine them with negations, as in “Did John not go to the store?” We can also generate commands, such as “Go to the store!” and polite requests, such as “Please go to the store.” Politeness is treated as syntax without semantics, so the last two sentences would have identical logical expressions. Having syntactically different but semantically identical language-logic pairs in our synthetic training data is essential for creating a system that can faithfully encode natural language into logic.

### 4.5 Grammar and agreement

To create our synthetic corpus, we developed software that builds a frame structure for each sentence. The components of the frame are a “maximal” sentence, with modal attributes possibly negated, around epistemics, possibly negated, around a subject-verb-object form, possibly negated, and each reflecting the possibility of being a group, a quantity, or an individual. While this results in a well-bounded algorithm for constructing grammatical agreement, it is arguably unnaturally constrained and ad hoc. In previous research (Cai et al., 2016), we attempted to use a statistical parser (Manning et al., 2014) to parse a sentence into universal dependencies (UD) (de Marneffe et al., 2021) and then use that as the basis for a translation to logic. However, the non-deterministic nature of the transformation of natural language to UDs meant that we did not have enough stability in the UD representations to create a reliable translator to logic. We could explore creating a UD parse as part of our language-logic training pair generation, because we have complete control over the generation, and UD would be just a data structure and markup language for linguistic elements.

Another challenge is that languages have many varied irregular morphological structures. To our knowledge, there is no electronically readable, broad coverage database of all of English morphology. In the past, collecting such information would often be the province of many graduate students, but the size of the language makes it prohibitively expensive to collect such data. LLMs, however, arguably have all the morphology of English embedded within them, and it should be possible to extract that knowledge. We have treated a set of different LLMs as though they were human linguistics students, asking them for data on the tenses, plurals, and collocations between verbs and prepositions (Thompson and Pease, 2026). With several such systems working on the same questions, we have used metrics for inter-subject agreement to assess whether their data is reliable. Once integrated into our synthetic training pipeline, this information should allow us to avoid many common grammatical errors in our training set.

### 4.6 Capturing common sense

We completed several iterations of generating and refining our training set. One method was to use the detailed types provided by SUMO to specify co-locational restrictions. We generated a sample of 6,000 sentences and asked student interns to mark them as to determine whether they were reasonable or not, and if not, what common-sense constraint was violated. Lastly, as time permitted, we, as researchers who are fully trained in SUMO, marked the frames with the SUMO type restrictions that would catch the problem in future-generated sentences.

For example, take the sentence “Who ferments the day duration.” Only an OrganicObject can be fermented, making this sentence nonsensical. Another example is “The concierge will not be drinking a game.” One drinks a Liquid, a Beverage, or possibly a Medicine. In both of these examples, a type constraint can be added to SUMO to prevent future generation of sentences with these sorts of semantic errors. A type constraint on the patient type of a process is defined as in (Listing 20). This constraint can then be used in formalizing the idea that only an OrganicObject can be fermented (Listing 21).

Listing 20 More Complex Type Restrictions


(domainSubclass patientType 1 Process) 
(domainSubclass patientType 2 Entity)
       
(=> 
  (and 
    (patientType ?P ?E) 
    (instance ?PI ?P) 
    (patient ?PI ?EI)) 
  (instance ?EI ?E))

Listing 21 Complex Type Statement


(patientType Fermentation OrganicObject)

However, type constraints are insufficient. “John puts the cup on the table.” is valid. One puts an Object in a PositionalAttribute to another object or Region. But “John puts the planet on the table.” Or “John puts the atom on the table.” is wrong because the relative scale of the objects is wrong. Even with thousands of classes that can specify type restrictions, we need a more expressive language for specifying restrictions. Fortunately, SUMO is written in a language that has exactly that sort of expressiveness. We can state that any object resting on a table is not more than 100 times the weight of the table. In the formalization below MeasureFn is a function that takes a RealNumber and a UnitOfMeasure and denotes a Quantity (Listing 22).

Listing 22 A Common Sense Restriction

 
(=> 
  (and 
    (instance ?T Table) 
  #x000A0; (weight ?T 
      (MeasureFn ?TW ?U))
    (weight ?O 
      (MeasureFn ?OW ?U)) 
    (orientation ?O ?T On))
  (not 
  (greaterThan ?OW 
    (MultiplicationFn ?TW 100))))

While many tables might still break under objects ten times their weight, we have now excluded many obvious nonsense sentences. It is also not nonsense to describe in a sentence a moment of poor judgment in which one puts something too heavy on a table and causes it to break.

While SUMO contains many common-sense constraints on how objects behave and interact in the world, it certainly does not contain all or most of such restrictions. This study has a further benefit beyond generating a corpus of training sentences, in that it makes clear which additional formulas are needed so that SUMO can reject many states of affairs that violate common sense.

### 4.7 Automated coherence filtering

Graduate students and student interns were enlisted to evaluate a sample of 6,000 generated sentences. Each sentence was marked as to whether it was completely sensible, pragmatically questionable, or had clearly the wrong type of subject, object, or indirect object. Slightly less than half (2,825 out of 6,000) were found to be completely sensible. For the sentences that had the wrong types, we asked the markers to suggest type restrictions with respect to SUMO types that would prevent a similarly incorrect sentence from being generated in the future. We used this set of labeled sentences to train a machine learning system to recognize coherent and incoherent sentences.

At the scale of millions of sentences, manual filtering is infeasible, motivating the need for automated coherence discriminators. Type constraints and common-sense rules in SUMO will reduce this ratio, yet a fraction of synthetically generated sentences remains linguistically incoherent. This problem and our approach to solving it have been previously reported and are summarized in this section (Thompson et al., 2026).

Training datasets with higher semantic coherence have been shown to improve LLM performance (Roberts et al., 2019), so our objective is *high-precision filtering*: automatically selecting a subset of sentences that is highly likely to be coherent. Since synthetic sentence generation is computationally inexpensive, we accepted a high false negative rate to minimize the incidence of incoherent sentences in the training corpus.

Three LLM-based filtering methods were evaluated on approximately 6,000 human-labeled sentences. The first method extracted token-level surprisal statistics from a pretrained LLaMA 3.2 model (Touvron et al., 2023) and trained three classifiers, Logistic Regression (LR), Random Forest (RF), and Support Vector Machine (SVM) (Akhilesh and Sabitha, 2025), using features such as mean negative log-likelihood, high-percentile surprisal, and token repetition rates. Decision thresholds for each classifier were calibrated using the Wilson score lower confidence bound on precision (McGrath and Burke, 2021), providing conservative guarantees even when the number of accepted sentences was small. The second method submitted each sentence to LLaMA 3.2 via a prompt requesting a binary coherence judgment. The third method was a cascade that used the computationally cheaper LR discriminator as a pre-filter before applying the more expensive prompt-based discriminator.

Results showed a tradeoff between precision and the fraction of coherent sentences retained. Among the expected token classifiers, LR achieves the highest precision of 0.77 and the lowest leakage rate. The prompt-based discriminator improves precision to 0.905 but reduces throughput. Both cascade variants achieve perfect precision on the held-out test set, though at a substantially reduced acceptance rate. A diversity analysis confirms that high-precision filtering does not collapse the corpus into short, repetitive sentences, as the type-token ratio and TF-IDF pairwise similarity remain healthy across all discriminators.

Beyond improving training data quality, the coherence filtering process yielded a useful secondary benefit: incoherent sentences flagged by the filters help identify gaps in SUMO's common-sense axioms. For example, the sentence “The concierge will be drinking a game.” reveals the absence of a type constraint requiring that the patient of Drinking be a Liquid. Each such case provides an actionable signal for ontologists to add new common-sense axioms, thereby improving both sentence generation and later reasoning.

### 4.8 Dataset distribution

**Figure 2:**
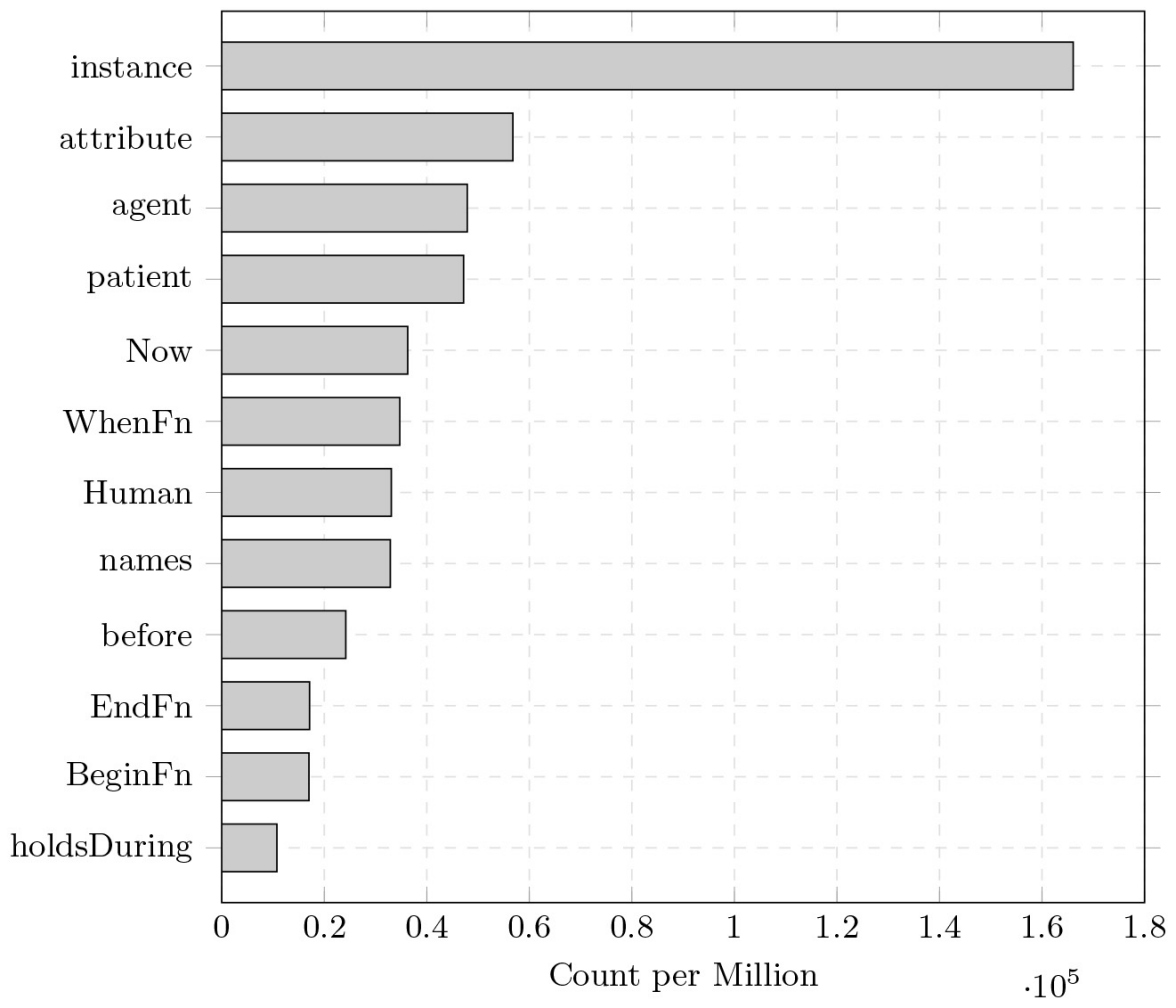
Count per million of the twelve most common SUMO terms in the dataset.

**Figure 3:**
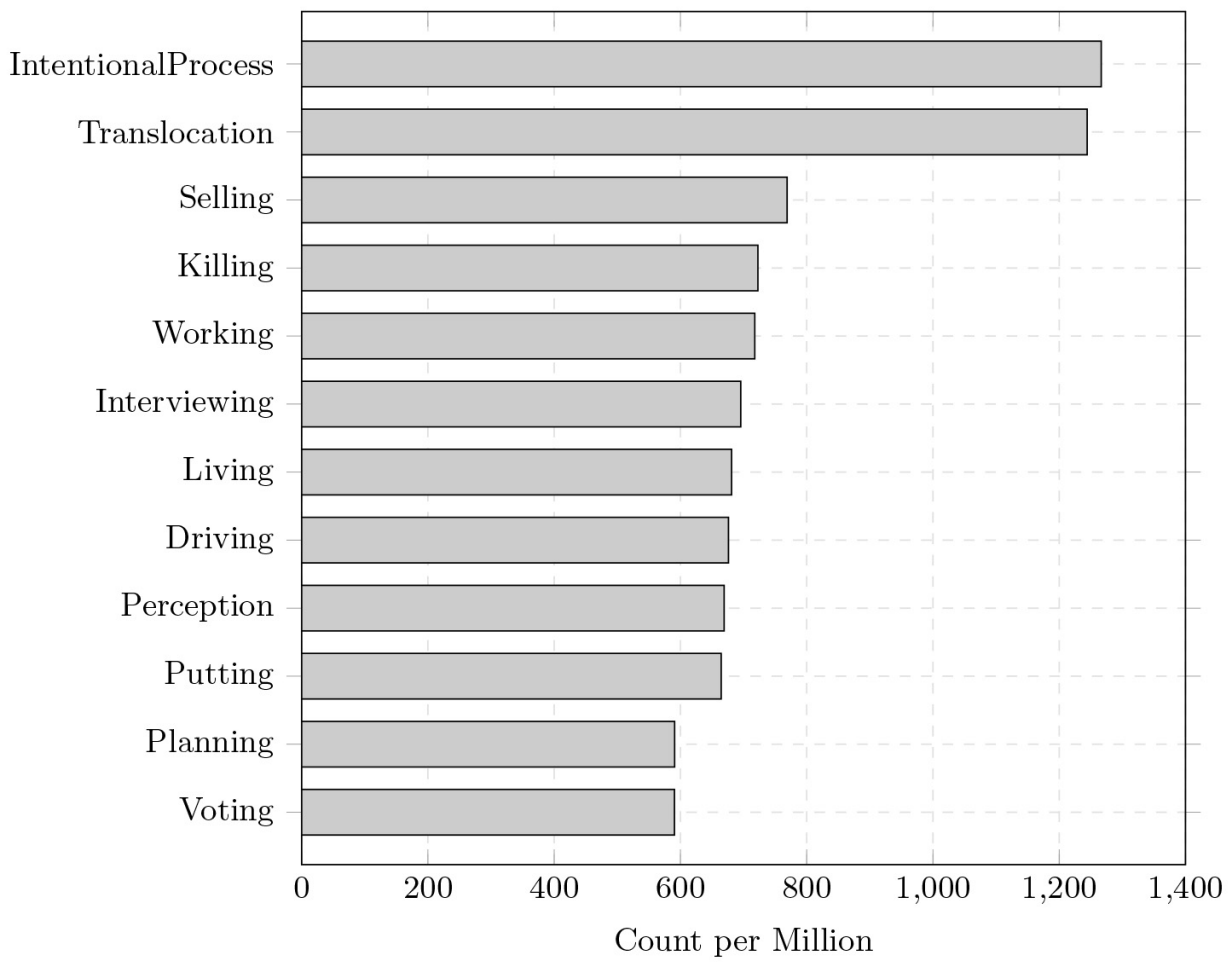
Count per million of the twelve most common SUMO processes in the dataset.

**Figure 4:**
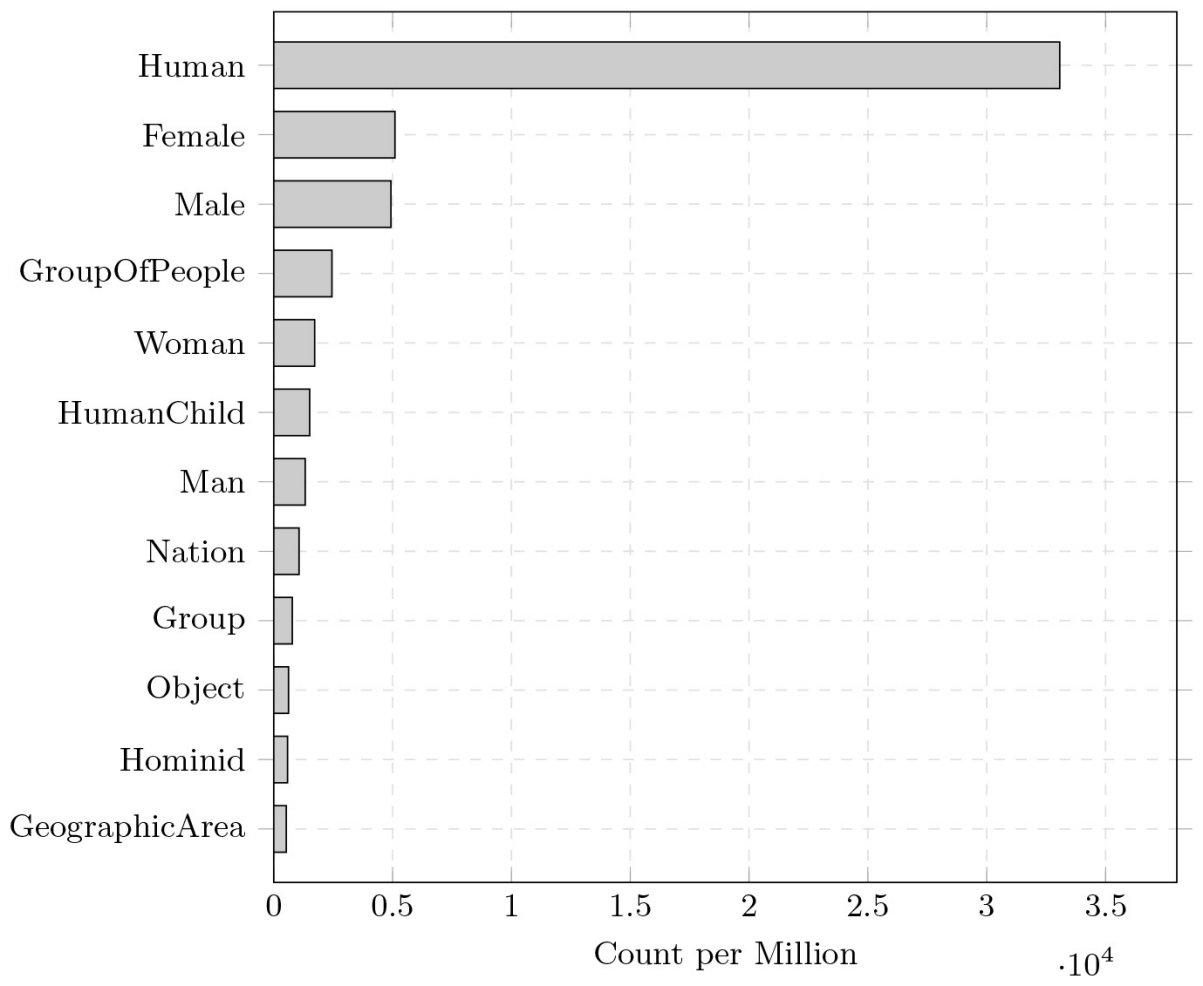
Count per million of the twelve most common SUMO object subclasses in the dataset.

We generated a dataset of 6.8 million sentences and their logical equivalents using the methods described above. While all three generation methods presented in Section 4 were used, the majority were generated using a compositionality approach (approximately 6 million). The distributions of formal SUMO terms are shown in Figures 2–4.

By far, the most common terms are those associated with instantiation, tense, and process case roles. The most common processes are the somewhat generic IntentionalProcess and Translocation. The remaining processes each occur fewer than 1,000 times in a million sentences and show little variance. By far, the most common SUMO objects used in SUMO are human-agent-related. The superclass Human appears in approximately 35,000 logic formulas. Because humans are disproportionately common agents in many processes, this skew is both expected and desired.

### 4.9 Model training

Frontier models use publicly available data for training. In the long term, probably, the most significant impact of English/logic pairs generated here will be on the ability of frontier models to perform SUO-KIF translations. However, numerous model training iterations were conducted throughout this research project. Early in the research, training was conducted with the Flan-T5 base model (Longpre et al., 2023), downloaded from the Hugging Face repository.^9^ All training on both models was conducted using 3 NVIDIA L40S GPUs built on the Ada Lovelace architecture. Each GPU has 46 GB of VRAM. There were 24 CPUs and 240 GB RAM allocated to the job. Training took approximately 17 h.

The data was randomly shuffled and split 80% training and 20% validation. The validation data set was kept completely separate, with model weights updated only through interaction with the training set. Training ran for 5 epochs, where an epoch is a full pass through the entire training dataset. After each epoch, the model was evaluated on the validation set for informational purposes and to chart training progress. The AdamW optimizer (Loshchilov and Hutter, 2017) was used as the gradient descent algorithm. Gradient accumulation batch sizes of 256 were used (i.e., 256 samples were observed before updating weights).

An illustrative training run produced 6.5 million English-logic pairs. After the first epoch, the model nears convergence, and further training shows minimal improvement. After each epoch, the model was evaluated on the validation set. Improvement after the first epoch was slight, remaining near the 77% exact match rate. Some outputs produced correct logic, but with different (but consistent) variable names. Accounting for this, the output correctness on the validation set on the final epoch was 81.4%. Syntax was valid in 99.99% of the validation set, meaning that the SUO-KIF returned by the model parsed correctly.

Outputs from the validation set were also automatically checked for hallucinated SUMO terms. Each term was extracted from the output and verified to be present in the SUMO ontology. An output was considered valid if the formula contained no hallucinated terms whatsoever. In the validation set, 99.46% of lines contained no hallucinated terms.

## 5 Overall system description

While this paper focuses on generating the synthetic L2L training set, an overview of the end-to-end language understanding system helps understand the utility of the training set.

**Figure 5:**
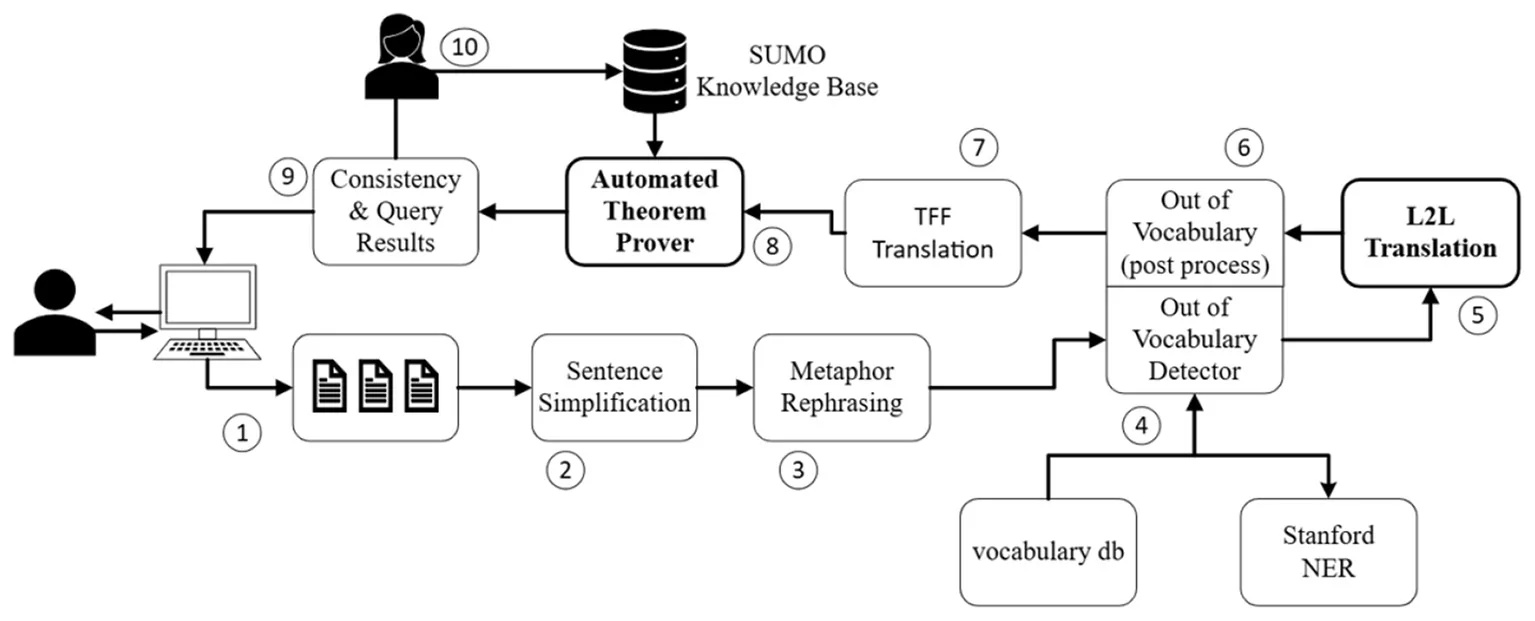
Data flow diagram for HyNOLU.

The Hybrid Neuro-Ontological Language Understanding (HyNOLU) system was designed to handle a variety of sentences, including those with a broad range of grammatical structures, extensive vocabulary, and metaphors. Once sentences are translated to logic, they can be used by ATPs. The block diagram of the dataflow at runtime is shown in Figure 5.

The specific points in the block diagram are explained below. An example is used to illustrate how the system operates. The example is drawn from a fictitious but representative news article about a weather event in Florida and is translated into logic statements.

1. A sentence from the input document is extracted from the article, for example: “The nightmare Hurricane Milton destroyed the beautiful bell pepper crop in western Florida, and left many residents homeless.”
2. The sentence is simplified with an LLM, and co-references are resolved with the Stanford Stanza library. Example: “The nightmare Hurricane Milton destroyed the beautiful bell pepper crop in western Florida. Hurricane Milton left many residents homeless.”
3. The sentence is analyzed for the presence of a metaphor. In this example, the hurricane is not literally a nightmare. The metaphor is replaced with a literal reading by use of prompts to an LLM. Example: “The very bad Hurricane Milton destroyed the beautiful bell pepper crop in western Florida. Hurricane Milton left many residents homeless.”
4. An out-of-vocabulary detector checks if any of the words in the sentence are not in our database of words that correspond to SUMO concepts, which consists of WordNet plus specialized lexical tokens from SUMO. If an unknown word or phrase exists in the sentence, it is replaced with a tag, using the Stanford Named Entity Recognizer (NER) (Qi et al., 2020) to identify the part of speech. In the example, it is determined that “Milton” is not in the database. Example: “The very bad hurricane  < UNK\_NOUN1> destroyed the beautiful bell pepper crop in western Florida. Hurricane  < UNK\_NOUN1> left many residents homeless.”
5. The L2L LLM translates the sentence into SUO-KIF and SUMO.
6. A post-processor restores out of vocabulary words.
7. The resulting SUO-KIF logic statements are translated into the TPTP language syntax.
8. Vampire is used to prove inconsistencies (if present) between the newly created logic statements and SUMO.
9. The results are processed for presentation to the user. Proofs are rewritten to be human-friendly, including proof graphs and a natural English representation.
10. The results are additionally returned to the domain expert. The domain expert makes one or more of several decisions as applicable:

- Reject the logic statements.
- Add the logic statements to the theory.
- If a contradiction is introduced by the newly added logic statements, choose which logic statements are more authoritative and purge the others from the theory.
- If a contradiction exists, keep both sets of logic statements and designate one or both as “beliefs” with modal logic.

(exists (?H ?D ?C ?BAD ?BEAUTIFUL) 
  (and 
    (instance ?H Hurricane) 
    (names ~<UNK_NOUN1>~ ?H) 
    (instance ?BAD SubjectiveStrongNegativeAttribute) 
    (attribute ?H ?B) 
    (instance ?D Destruction)
    (instance ?C Collection) 
    (memberType ?C SweetPepper) 
    (instance ?BEAUTIFUL SubjectiveStrongPositiveAttribute) 
    (attribute ?C ?BEAUTIFUL) 
    (patient ?D ?C) 
    (agent ?D ?H) 
    (eventLocated ?D
      (WesternFn FloridaUnitedStates))))


(exists (?H ?PEOPLE) 
  (and 
    (instance ?H Hurricane) 
    (names ~<UNK_NOUN1>~ ?H) 
    (instance GroupOfPeople ?PEOPLE) 
    (holdsDuring 
      (BeginFn 
        (WhenFn ?H)) 
      (=> 
        (and 
          (instance ?PERSON Human) 
          (member ?PERSON ?PEOPLE)) 
        (exists (?HOME) 
          (and
            (instance ?HOME PermanentResidence) 
            (possesses ?PERSON ?HOME)))) 
      (holdsDuring 
        (EndFn 
          (WhenFn ?H)) 
        (=> 
          (and
            (instance ?PERSON Human) 
            (member ?PERSON ?PEOPLE) 
            (instance ?HOME PermanentResidence)) 
          (not
            (possesses ?PERSON ?HOME)))))))


(exists (?H ?D ?C ?BAD ?BEAUTIFUL) 
  (and 
    (instance ?H Hurricane) 
    (names ~Milton~ ?H) 
    (instance ?BAD SubjectiveStrongNegativeAttribute) 
    (attribute ?H ?B) 
    (instance ?D Destruction) 
    (instance ?C Collection)
    (memberType ?C SweetPepper) 
    (instance ?BEAUTIFUL SubjectiveStrongPositiveAttribute) 
    (attribute ?C ?BEAUTIFUL)
    (patient ?D ?C)
    (agent ?D ?H) 
    (eventLocated ?D 
      (WesternFn FloridaUnitedStates))))


(exists (?H ?PEOPLE) 
  (and 
    (instance ?H Hurricane) 
    (names ~Milton~ ?H) 
    (instance GroupOfPeople ?PEOPLE)
    (holdsDuring 
      (BeginFn 
        (WhenFn ?H)) 
      (=> 
        (and 
          (instance ?PERSON Human) 
          (member ?PERSON ?PEOPLE)) 
        (exists (?HOME) 
          (and
            (instance ?HOME PermanentResidence) 
            (possesses ?PERSON ?HOME)))) 
      (holdsDuring 
        (EndFn 
          (WhenFn ?H)) 
        (=> 
          (and 
            (instance ?PERSON Human) 
            (member ?PERSON ?PEOPLE) 
            (instance ?HOME PermanentResidence)) 
          (not
            (possesses ?PERSON ?HOME)))))))

## 6 Results and discussion

This research demonstrated an effective pathway for creating a diverse dataset for later LLM training. Importantly, and a unique contribution, our dataset of diverse English sentences with logical equivalents is grounded in a formal ontology.

We previously reported (Thompson et al., 2025b) on the techniques used to train an automated language-to-logic translation system on our set of language-logic pairs, using the T5 transformer (Raffel et al., 2020) and custom tokenization. It appears that one million pairs is an optimal size, at least for sentences of our current scope and complexity, so that is what we currently disseminate (see section 7).

We have shown the ability to generate a large corpus of language-logic pairs, covering a large portion of the lexical semantics, if not the grammar, of English sentences. The primary challenge is to continue scaling this study so that it can be reliably used as an input method for logic statements that augment a comprehensive and expressive ontology.

Our current automatically generated language-to-logic corpus consists of one million paired statements of an English sentence and its logical equivalents, as stated in SUO-KIF, using concepts defined in SUMO. We have validated each logical expression for being consistent with SUO-KIF syntax and with the type restrictions in SUMO. We have further validated the sentences for “weirdness” as identified with a machine learning system trained on a set of sentences marked as normal or nonsensical by a team of human raters.

An additional next step is to test each formula in the corpus to determine whether it is consistent with the entire SUMO (Pease and Schulz, 2022). While the machine learning methods and simple type restrictions discussed above will catch some nonsensical sentences, employing theorem proving is needed to capture deeper issues, such as those described by the formula in Listing 22. A challenge is that while table lookups for type restrictions are very fast, theorem proving may take from hundreds of milliseconds to hundreds of seconds when a set of formulas as large as SUMO is used (Pease et al., 2010), and there is no guarantee of completion, so generous timeouts must be used. To handle this more demanding computation, we are using a computing cluster to continuously run theorem proving on our generated logic statements, accumulating a set of fully validated formulas.

While research remains to improve the common-sense restrictions in the SUMO and to apply them to generate corpora with improved realism, the current set can be productively used to train machine learning systems to translate language into logic and to expand the set of people capable of adding knowledge to SUMO.

## Data Availability

The datasets presented in this study can be found in online repositories. The names of the repository/repositories and accession number(s) can be found in the article/supplementary material.

## Footnotes

1
www.ontologyportal.org

2
https://github.com/ontologyportal/sigmakee/blob/master/suo-kif.pdf

3
http://sigma.ontologyportal.org:8080/sigma/Browse.jsp

4 Sutcliffe, G. (2026). ShZZaM: An LLM+ATP Natural Language to Logic Translator. Personal communication. doi: 10.32473/flairs.39.1.141689

5
http://sigma.ontologyportal.org:8080/sigma/Browse.jsp?kb=SUMO&lang=EnglishLanguage&kb=SUMO&term=Object

6
http://sigma.ontologyportal.org:8080/sigma/Browse.jsp?lang=EnglishLanguage&kb=SUMO&term=Artifact

7
https://wordnet.princeton.edu/documentation/wninput5wn

8
http://sigma.ontologyportal.org:8080/sigma/Browse.jsp?kb=SUMO&lang=EnglishLanguage&kb=SUMO&term=CaseRole

9 Model can be found at https://huggingface.co/google/flan-t5-base.
